# Smash and DASH with Cas9

**DOI:** 10.1186/s13059-016-0905-4

**Published:** 2016-03-05

**Authors:** Vijay Ramani, Jay Shendure

**Affiliations:** Department of Genome Sciences, University of Washington, 3720 15th Ave NE, Seattle, WA 98195 USA; Howard Hughes Medical Institute, 3720 15th Ave NE, Seattle, WA 98195 USA

## Abstract

DeRisi and colleagues present a creative application for Cas9 in vitro, using it to deplete unwanted sequence from DNA libraries. It seems plausible that the in vitro use of CRISPR/Cas9 has unrealized potential to revolutionize the practice of molecular biology well beyond genome editing.

## Cas9 as a molecular biological tool

Programmed cleavage of DNA by clustered regularly interspersed palindromic repeats (CRISPR)/Cas9 has been a transformative development in biomedical research [1]. Short guide RNA (sgRNA) libraries have quickly overtaken small hairpin RNA (shRNA) libraries as the de facto functional genomics tool, and the ease with which Cas9 enables genome perturbation both in single clones and in high-throughput is revolutionizing the way biologists approach their craft [2]. However, the potential utility of Cas9 as a more general reagent for molecular biology has received far less attention.

In this issue of *Genome Biology*, DeRisi and colleagues describe depletion of abundant sequences by hybridization — or DASH — a novel molecular technique that employs the inherent programmability of the Cas9 nuclease to perform in vitro depletion of unwanted library molecules prior to PCR amplification [3]. The authors provide proof-of-concept for this method, applying it in the contexts of massively parallel sequencing and digital droplet PCR (ddPCR) experiments.

## DASHing away unwanted molecules

Though massively parallel sequencing costs continue to decrease rapidly, the need to maximize sequencing coverage of regions of interest ever remains a challenge. This is particularly true for molecular counting applications (for example, RNA-seq) where sensitivity is often important, yet abundant unwanted sequences like ribosomal RNA (rRNA) and small nucleolar RNAs (snoRNAs) comprise a large fraction of sampled molecules. There are currently several approaches for addressing this issue, nearly all of which rely on hybridization of sequence-specific oligonucleotides to target sequences. For example, RNase H-based methods for rRNA depletion use the heteroduplex-specific RNase H to digest away RNA–DNA hybrids in a targeted way, but are limited to RNA applications. Hybrid capture and subtractive hybridization, which employ streptavidin pull-down of biotinylated hybridization probes [4], are applicable to any single-stranded or double-stranded library of DNA molecules, but are time-intensive, typically require large amounts of starting material, and are expensive when purchased as kits.

The DASH method presents a novel, robust alternative to the current suite of tools for targeted sequence depletion. In DASH, sgRNA libraries tiling over undesired regions are designed and T7-transcribed in vitro from a pool of double-stranded DNA templates. Cas9 ribonucleoprotein complexes are then assembled and incubated with DNA libraries, digesting unwanted molecules and preventing them from acting as viable PCR templates.

The authors demonstrate DASH in three contexts: first, to deplete mitochondrial rRNA sequence in low-input RNA-seq libraries from HeLa total RNA; second, to deplete mitochondrial rRNA sequence to aid sequence-based pathogen detection in patient cerebrospinal fluid (CSF); and third, to increase the relative abundance of a known driver *KRAS* mutation with respect to wild-type sequence in a synthetic genomic DNA (gDNA) mixture. The efficiency of DASH in each context is striking. In HeLa libraries, mitochondrial rRNA contamination dropped from a 61 % to a miniscule 0.055 % of uniquely mapped sequencing reads. In libraries generated using CSF from a patient with meningoencephalitis, the authors observed a twofold to fourfold increase in pathogen-specific reads, enabling more sensitive detection of infectious agents. Finally, when using DASH to deplete wild-type *KRAS* sequence in synthetic gDNA mixtures prior to molecule counting on a ddPCR platform, the authors observed 10- to 65-fold enrichment for the mutant *KRAS* allele of interest, further highlighting the versatility of their approach.

A common worry with many Cas9 applications is the relative fidelity of the nuclease, particularly in cases where vast molar excesses of enzyme are used with respect to targets [5]. In this regard, one encouraging result presented by the authors is the relatively small effect of off-target cleavage when using DASH. While this is likely as much a function of the gRNAs selected as it is a general feature of the protocol itself, it is reassuring that when analyzing DASHed HeLa RNA-seq data, the authors only detected one off-target, which harbored strong identity to a region of the 16S mitochondrial rRNA gene. This suggests that with optimal gRNA design parameters, users should not expect gross overdigestion of DNA libraries due to Cas9 off-targets.

## Future applications for DASH and Cas9 in vitro

DASH represents a simple, flexible approach for depleting practically any sequence or sequences in DNA libraries — an approach that is less costly in terms of reagent price and raw bench time with respect to other methods, particularly when users supply their own purified Cas9. Given this, we anticipate that DASH will find a host of other applications. ATAC-seq [6] and single-cell ATAC-seq [7, 8] libraries are notorious for contamination by mitochondrial gDNA, which can comprise as many as 70 % of sequencing reads. DASHing away unwanted reads using a tiling library of mitochondrial genome-specific sgRNAs seems a potentially straightforward solution to minimizing these unwanted reads. DASH could also facilitate the efficiency of single cell RNAseq — abundant polyadenylated transcripts (for example, GAPDH, actin) could be depleted from libraries, enabling higher coverage of other transcripts of interest across large numbers of cells.

We are also intrigued by the myriad other potential applications for in vitro Cas9 digestion. While the authors chose to focus on using Cas9 as a reagent for depletion, their experiments to deplete wild-type *KRAS* alleles suggest other possibilities. For example, by generating libraries containing guides overlapping known polymorphisms one could use in vitro Cas9 digestion to enrich for sequences in a haplotype-specific manner. Also, by taking advantage of decreasing oligosynthesis costs [9], one could conceivably multiplex DASH-like experiments, using pools of many thousands of gRNAs per experiment to precisely cleave and enrich for desired sequences at scale.

In vitro Cas9 applications need not necessarily involve digestion. Recent work has suggested that nuclease-dead Cas9 variants (dCas9) can be used in cells to enrich for genomic regions through a chromatin immunoprecipitation assay termed enChIP [10]. While speculative, one could imagine in vitro experiments where epitope-tagged dCas9 is pulled-down following incubation with sequencing libraries; such an assay could be used for sequence enrichment, for example in instances where genomic insertion events (for example, retrotransposition, lentiviral integration) must be mapped.

## Closing remarks

A more general point is that restriction enzymes are utterly central to molecular biology (for instance cloning), but their range and specificity are inherently limited by the repertoire of what has evolved in microorganisms. In vitro Cas9 digestion remediates this through complete programmability of cleavage, a fact that has received too little billing given excitement about genome editing. As such, the application space pioneered by DeRisi and colleagues has the potential to revolutionize how we practice molecular biology on a daily basis.

## Abbreviations

CRISPR: Clustered regularly interspersed palindromic repeats
CSF: Cerebrospinal fluid
DASH: Depletion of abundant sequences by hybridization
dCas9: Nuclease-dead Cas9
ddPCR: Digital droplet PCR
gDNA: Genomic DNA
rRNA: Ribosomal RNA
sgRNA: Short guide RNA
shRNA: Short hairpin RNA
snoRNA: Small nucleolar RNA
